# Biodegradable film: a sustainable alternative to polyethylene film for Loess Plateau maize production

**DOI:** 10.3389/fpls.2026.1789837

**Published:** 2026-03-11

**Authors:** Xiaoyu Sun, Zhihui Hu, Huihui Ding, Pengpeng Chen, Minhua Yin, Hongxiang Hu, Xiaobo Gu, Yuannong Li, Heng Fang

**Affiliations:** 1 Anhui Province Key Lab of Farmland Ecological Conservation and Nutrient Utilization, College of Resources and Environment, Anhui Agricultural University, Hefei, China; 2 College of Water and Architectural Engineering, Shihezi University, Shihezi, China; 3 College of Water Conservancy and Hydropower Engineering, Gansu Agricultural University, Lanzhou, China; 4 Key Laboratory of Agricultural Soil and Water Engineering in Arid and Semiarid Areas of Ministry of Education, Northwest A&F University, Yangling, China

**Keywords:** evapotranspiration, reactive nitrogen emissions, soil nitrogen, water– and nitrogen use efficiency, yield

## Abstract

The use of biodegradable film as an alternative to polyethylene film is still controversial. Thus, a split-plot field trial was performed with planting patterns [traditional planting (FNM), and ridge-furrow planting with polyethylene and biodegradable films mulching (RPM, RBM)] coupled with nitrogen application levels [0 kg ha^−1^ (N0), 180 kg ha^−1^ (N180)] to explore the substitution of RBM for RPM in maize production. Compared with FNM, RBM and RPM increased the soil moisture storage by 11.6% and 11.5%, respectively, and soil mineral N concentration by 23.0% and 16.0% (under N180), but they reduced evapotranspiration by 3.9–11.7% and 7.9–36.4%, NH_3_ volatilization by 38.3% and 35.3%, and N_2_O emissions by 69.4% and 82.3% (2019 season). Moreover, compared with FNM, RBM and RPM increased the leaf area index by 16.8% and 19.4%, and aboveground dry matter by 10.8% and 16.5%, respectively. However, both mulching and N fertilization reduced the soil organic matter after 3 years of production. Path analysis revealed the differences in the influencing pathways of yield and the utilization of water and nitrogen. Consequently, compared with FNM, RBM and RPM under N180 improved the maize yield by 6.2% and 8.4%, and water use efficiency by 17.3% and 44.4%, respectively, but regardless of fertilization, they increased N harvest index by 9.2% and 10.8% and N use efficiency (2019 season) by 6.5% and 4.0%. These results demonstrated that the biodegradable film mulching was a viable replacement for polyethylene film mulching in maize production on the Loess Plateau in terms of soil fertility, plant growth, yield, and utilization of water and nitrogen.

## 1 Introduction

The Loess Plateau, a major rainfed agricultural region in China, accounts for about 9.1% of the total national grain yield (Chen et al., 2022). Thus, increasing or ensuring the crop yield in the Loess Plateau is of vital importance for safeguarding state food security. Grain maize (*Zea mays* L.) is one of the main cereal crops on the Loess Plateau, which requires a large amount of water. However, the combination of low and inhomogeneous precipitation and significant evaporation during the crop season hinders the development of local agriculture (Fang et al., 2021a; Han et al., 2021). Moreover, this issue has been further aggravated by global climate change in recent years, with more frequent extreme weather (Guo et al., 2024). Therefore, implementing water-saving practices is crucial for improving or maintaining grain yield.

Film mulching effectively prevents the interchange of moisture and gases between soil and air, and it is widely adopted in crop production (such as in maize, rapeseed, and tomato) in arid and semi-humid drought-prone regions due to its beneficial effects on retaining heat, maintaining soil moisture, increasing crop productivity, and providing ecosystem services (Zhou et al., 2020; Wang et al., 2022; Xu et al., 2024, 2025). To fully utilize limited precipitation, mulching is often coupled with the planting method of ridge-furrow (RFFM), which collects precipitation and promotes its infiltration (Gan et al., 2013). Previous research indicates that RFFM increases the yield, biomass, water– and nitrogen use efficiency (WUE, NUE), harvest index, economic benefits, and precipitation utilization efficiency, compared with traditional planting without mulching (Chen et al., 2022; Lai et al., 2024; Wang et al., 2025). However, the residual polyethylene film and consequent microplastic pollution have become an increasingly prominent problem, so biodegradable film is widely studied as an ideal replacement for polyethylene film in recent years due to its degradability.

Previous studies that compared biodegradable film (RBM) and polyethylene film (RPM) under RFFM mainly focused on the soil properties (especially soil moisture), yield, and resource utilization efficiency (especially WUE) (Fang et al., 2022a; Lai et al., 2024; Shangguan et al., 2024; Xiong et al., 2025). Some studies also explored mechanisms related to increases in the production and efficiency under RFFM. For instance, Yang et al. (2024) reported that RPM noticeably improved the contents and stratification ratios of soil nutrients (mineral and total N, P, K, and soil organic carbon) at soil layers of 0–40 cm and 0–60 cm, respectively, compared with traditional planting. Moreover, Xiong et al. (2025) found that maize yield increased under RPM was generally due to the higher soil temperature and promotion of nutrient absorption by plants, jointly enhancing the leaf photosynthetic efficiency. Lai et al. (2024) found that the yield differences under different mulching practices could be directly explained by variations in N uptake at post-silking and N remobilization at pre-silking. Fang et al. (2022b) indicated that compared with no mulching, RBM and RPM significantly showed the soil mineral N (SMN) content, maize N adsorption, soil moisture storage (SWS), yield, WUE, and N utilization indexes in maize, but decreased the water consumption and reactive nitrogen emissions. In general, agronomic practices impact plant growth by influencing soil properties to ultimately affect crop yield and resource utilization (Jin et al., 2007; Jia et al., 2019). Nevertheless, it is unclear how RBM and RPM increase the maize yield and resource utilization efficiency. Furthermore, due to differences in the compositions of biodegradable films and the environments where they are applied (Gu et al., 2020; Zhang et al., 2024), it is challenging to clarify the mechanisms that might affect whether biodegradable film can replace polyethylene film.

Therefore, we hypothesized that RFFM could improve the maize yield and water-nitrogen utilization, but the complementary effects of RBM and RPM on soil fertility and plant growth could also allow RBM to replace RPM. In the present study, a multi-year field trial was performed to test the response of maize to mulching patterns combined with N fertilization rates in order: (1) to assess combined impact of mulching and N fertilization on soil fertility and reactive N emissions, and maize growth and water-nitrogen utilization; and (2) to elucidate how RPM and RBM might enhance yield and resource utilization, and evaluate the possibility of using RBM as a replacement for RPM.

## 2 Materials and methods

### 2.1 Site description

Maize field trials were performed in 2018–2020 at the Key Laboratory of Agricultural Soil and Water Engineering in Arid and Semiarid Areas, Northwest A&F University (34°18′N, 108°24′E). The site has a climate of sub-humid and drought-prone, and weather data were supported by the Yangling National Meteorological Observation Station. The soil in this site was medium loam, and the content of organic matter, available nitrogen, available phosphorus, and available potassium was 16.9 g kg^−1^, 107.1 mg kg^−1^, 23.3 mg kg^−1^, and 88.5 mg kg^−1^, respectively.

### 2.2 Experimental design

The split-plot field trial was a completely randomized design, where the main plots were flat planting without mulching (FNM), RBM, and RPM. The sub-plots tested nitrogen application at rates of 0 kg N ha^−1^ (N0) and 180 kg N ha^−1^ (N180). The sub-plot, with three replicates, each measured 7.0 m × 4.0 m (28.0 m^2^). In addition, spacings of 1.0 m and 2.0 m were left between each plot and around the field, respectively. The width of the ridge and distance between adjacent ridges were 0.4 m and 0.6 m, respectively.

After rotary tillage, ridges were formed on the plot and covered with film before applying fertilizer. The biodegradable and polyethylene films exhibited optical transparency with a uniform thickness of 0.008 mm, and were provided by Yangling Ruifeng Environmental Protection Technology Co., Ltd. (Yangling, Shaanxi, China). The main components and degradation status of biodegradable film could be found in Fang et al. (2022b) and Fang et al. (2021a). The nitrogen fertilizer (urea, N≥46%), 120 kg ha^−1^ of phosphate fertilizer (P_2_O_5_≥16%), and 60 kg ha^−1^ of potash fertilizer (K_2_O≥51%) as basal fertilizer were manually spread out and raked into the soil. The cultivar Zhengdan 958 was manually planted on June 10–15 with a spacing of 60 cm × 30 cm. No irrigation was applied, and field management was conducted according to local practices.

### 2.3 Measurements and methods

#### 2.3.1 Soil moisture, evapotranspiration, and WUE

The content of soil gravimetric moisture was observed at a soil depth of 0–100 cm in the stages of jointing, anthesis, and grain filling, and at 0–200 cm before sowing and at harvest. Three representative samples were collected from the furrow/adjacent rows in the middle of plot with an auger, and each was separated into two parts. The first part was used as a fresh sample to determine the content of soil gravimetric water with the method of oven-drying, and the second part was air-dried and used for SMN analysis. The SWS (mm), evapotranspiration across the season (ET, mm), and WUE (kg ha^–1^ mm^–1^) via Equations 1, 2, and 3 respectively:

(1) $$\text{SWS}=\sum \left(SWC_{i}\times \gamma_{i}\times h_{i}\right)$$

(2) $$\text{ET}=P+SWS_{PS}-SWS_{PH}-D-R$$

(3) WUE=Yield/ET

where *SWC_i_* is soil water content in the ith soil layer, $\gamma_{i}$ is soil bulk density (g cm^–3^), $h_{i}$ is the depth of each soil layer (cm), *P* is the precipitation (mm), *SWS_PS_* and *SWS_PH_* are the soil moisture storage at pre-sowing and harvest (mm), respectively. *D* and *R* are the amount of deep drainage and runoff (mm), respectively, which were both zero due to the absence of long-term and heavy rainfall, and the ridges around plots.

#### 2.3.2 SMN and organic matter

The soil samples were extracted with 2 M KCl before determining the SMN content using the colorimetry method and Equation 4. After harvest, the soil organic matter (SOM) content in a soil depth of 0–40 cm was obtained by using the method of wet redox titration (Cheng et al., 2024). SMN was calculated as:

(4) $$\text{SMN}=\sum \left(c_{i}\times \gamma_{i}\times h_{i}\right)$$

where SMN and $c_{i}$ are the soil mineral N content and the mineral N concentration in the ith soil layer (kg ha^−1^), respectively.

#### 2.3.3 Reactive nitrogen emissions

The NH_3_ and nitrous oxide (N_2_O) emissions were observed with the methods of ventilation (Wang et al., 2004) and static closed chamber-gas chromatography (Zheng et al., 2008), respectively. The static closed chamber consisted of two primary components: a stainless-steel frame with a groove (40 × 40 × 10 cm) and a stainless-steel chamber (40 × 40 × 40 cm). The details of devices and operating procedures for NH_3_ and N_2_O collection were as described by Fang et al. (2022b). NH_3_ volatilization and N_2_O emissions were monitored daily in the first week post-fertilization and then at intervals of 2–3 days in the second week, 5–7 days in weeks 3–4, and 15–20 days subsequently until the harvest. NH_3_ and N_2_O emissions were also collected after rainfall. The amounts of NH_3_ volatilization and N_2_O emissions (kg ha^−1^) were calculated via Equations 5 and 6, respectively:

(5) $${\text{NH}}_{3}\text{ flux}=AN/(S\times t)\times f$$

(6) $${\text{N}}_{2}{\text{O}}_{\text{flux}}=\rho \times h\times \frac{dy}{dt}\times \frac{273}{273+T}\times f$$

where AN is the NH_3_ trapped in phosphoglycerol sponges (kg), S is the footprint area of the device (ha), *t* is the duration per capture event (d), *f* is the ratio of bare soil in plot, *ρ* is the standardized density of N_2_O (kg m^–3^), *h* is the effective height of the device (m), *dy*/*dt* is the change rate of N_2_O concentration, and *T* is the air temperature in chamber (°C).

#### 2.3.4 Plant growth, grain yield, and nitrogen utilization

During the seasons, three plants were obtained per plot at intervals of 15–20 days. The green leaf dimensions [length (L), width (W)] were measured using a rule to calculate leaf area index (LAI; Equation 7). Plants were separated and oven dried at 105°C for the first 0.5 h and to a stable weight at 75°C. The samples were then weighed, ground, and digested with H_2_SO_4_-H_2_O_2_ to determine the N content with a flow analyzer (AutoAnalyzer-III, SEAL Company, Germany). Then NUE and N harvest index (NHI) were derived with Equations 8–10. At maturity, ears were harvested from plants per plot and air-dried to obtain standard grain yield (standardized to 14% water content).

(7) LAI=0.75×n×∑(L×W)/A

(8) $$\text{PNU }=\;\sum \left(n_{i}\times M_{i}\right)$$

(9) NUE=Yield/PNU

(10) $$\text{NHI }=N_{g}\;/\;PNU$$

where A is the plot area (m^2^), n is the plant density per plot (plants ha^–1^), and *PNU* is the plant nitrogen uptake (kg ha^–1^). $n_{i}$ and $M_{i}$ are the nitrogen content (kg kg^–1^) and dry weight (kg ha^–1^) of plant organs, respectively. $N_{g}$ is the amount nitrogen of grain.

### 2.4 Statistical analysis

Data were processed with Microsoft Excel (Microsoft Corporation, Redmond, WA, USA). SPSS 20.0 (SPSS Inc., Chicago, IL, USA) was adopted to perform analysis of variance and technique for order preference by similarity to ideal solution (TOPSIS) to assess the effect of RBM and RPM on maize production. Significant effects among treatments were analyzed using Tukey’s HSD test at *P* < 0.05, and the TOPSIS analysis was conducted based on Liang et al. (2025). The method of Duncan’s new multiple range test (SSR) was used for multiple comparisons (*P ≤* 0.05). Structural equation modeling (SEM) (Zhou et al., 2019) was applied to explore the pathways of film mulching on maize production in terms of the soil properties and maize growth using the R (R version 4.1.1) package lavaan. OriginPro 2022 (Origin 9. 9, OriginLab Corporation, Northampton, MA, USA) was applied to conduct principal component analysis (PCA) and to create graphics.

## 3 Results

### 3.1 Meteorological conditions

**Figure 1:**
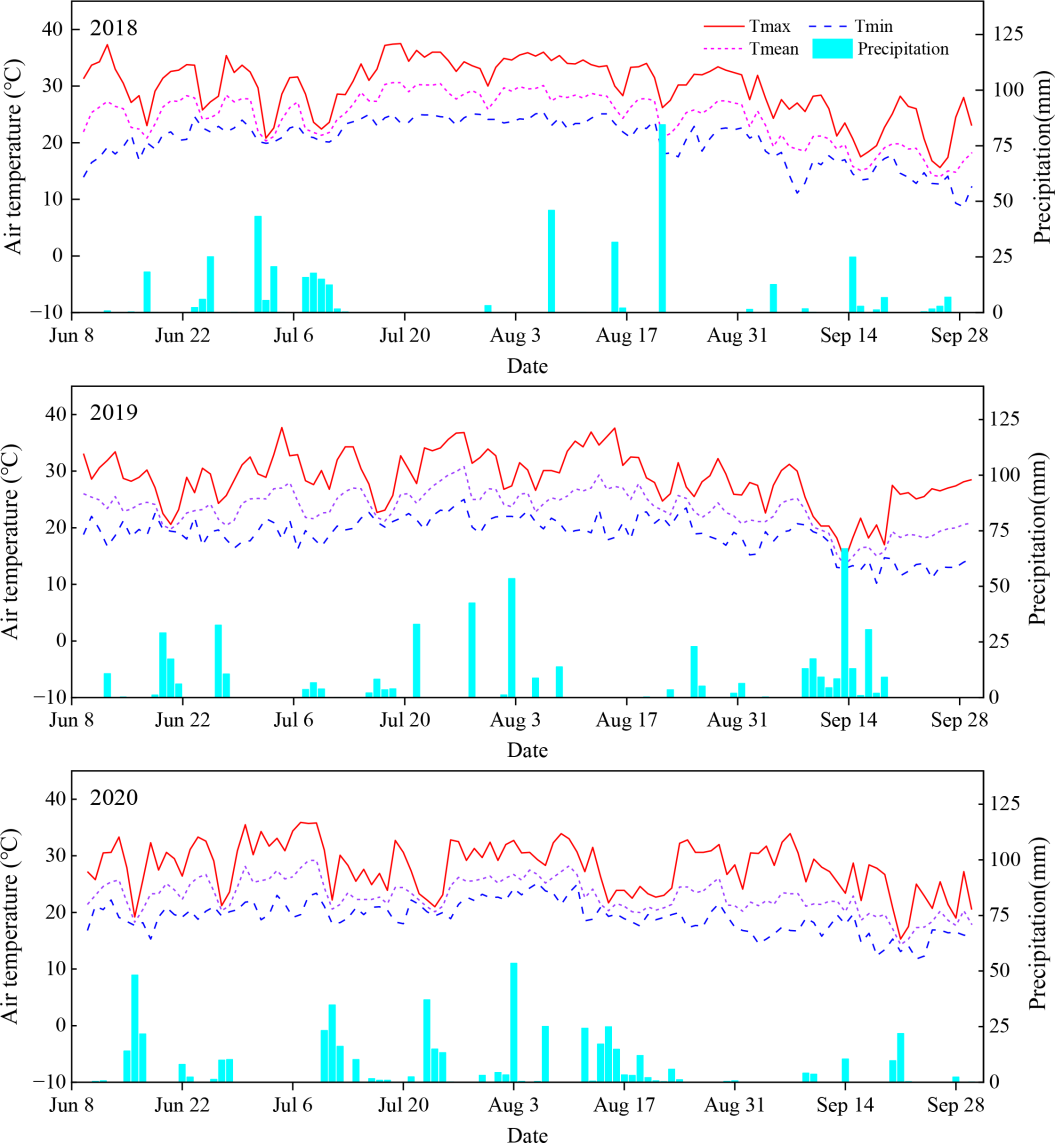
Meteorological conditions (daily air temperature and precipitation) during the maize growing seasons of 2018, 2019, and 2020. Tmax, Tmin, and Tmean were the maximum temperature, minimum temperature, and mean temperature, respectively.

The precipitation amounts during the seasons of 2018, 2019, and 2020 were 418.2 mm, 500.0 mm, and 530.0 mm, respectively (**Figure 1**). Furthermore, the precipitation was mainly distributed in July and August during the growing seasons of 2018 and 2020 when it accounted for 71.9% and 67.8% of the total precipitation, respectively, whereas it was mainly concentrated in August and September during the growing season of 2019 when it accounted for 58.8% of the total precipitation.

As for air temperature (**Figure 1**), the average maximum temperatures during the seasons in July, August, and September were 31.3°C, 32.9°C, and 24.3°C in 2018, 31.3°C, 30.5°C, and 24.3°C in 2019, and 29.4°C, 28.4°C, and 25.8°C in 2020, respectively. The mean minimum temperature during the seasons in July, August, and September was 23.0°C, 22.6°C, and 15.4°C in 2018, 20.6°C, 20.5°C, and 15.2°C in 2019, and 20.6°C, 20.6°C, and 16.0°C in 2020, respectively. The mean temperature during the growing seasons in July and August in 2018 was 1.1°C and 1.8°C higher, respectively, than the three-year average (25.5°C and 25.3°C), whereas the mean temperature in September was 0.5°C lower than the 5-year average (19.4°C). Furthermore, the mean temperature during the growing seasons in July and September in 2019 was consistent with the 3-year average (25.5°C and 19.4°C), whereas that in August was 0.3°C lower than the 3-year average. However, the mean temperature during the growing seasons in July and August in 2020 was 1.2°C and 1.5°C lower, respectively, than the three-year average, but 0.5°C higher in September than the 5-year average.

### 3.2 Soil water storage and content of mineral nitrogen and organic matter

**Figure 2:**
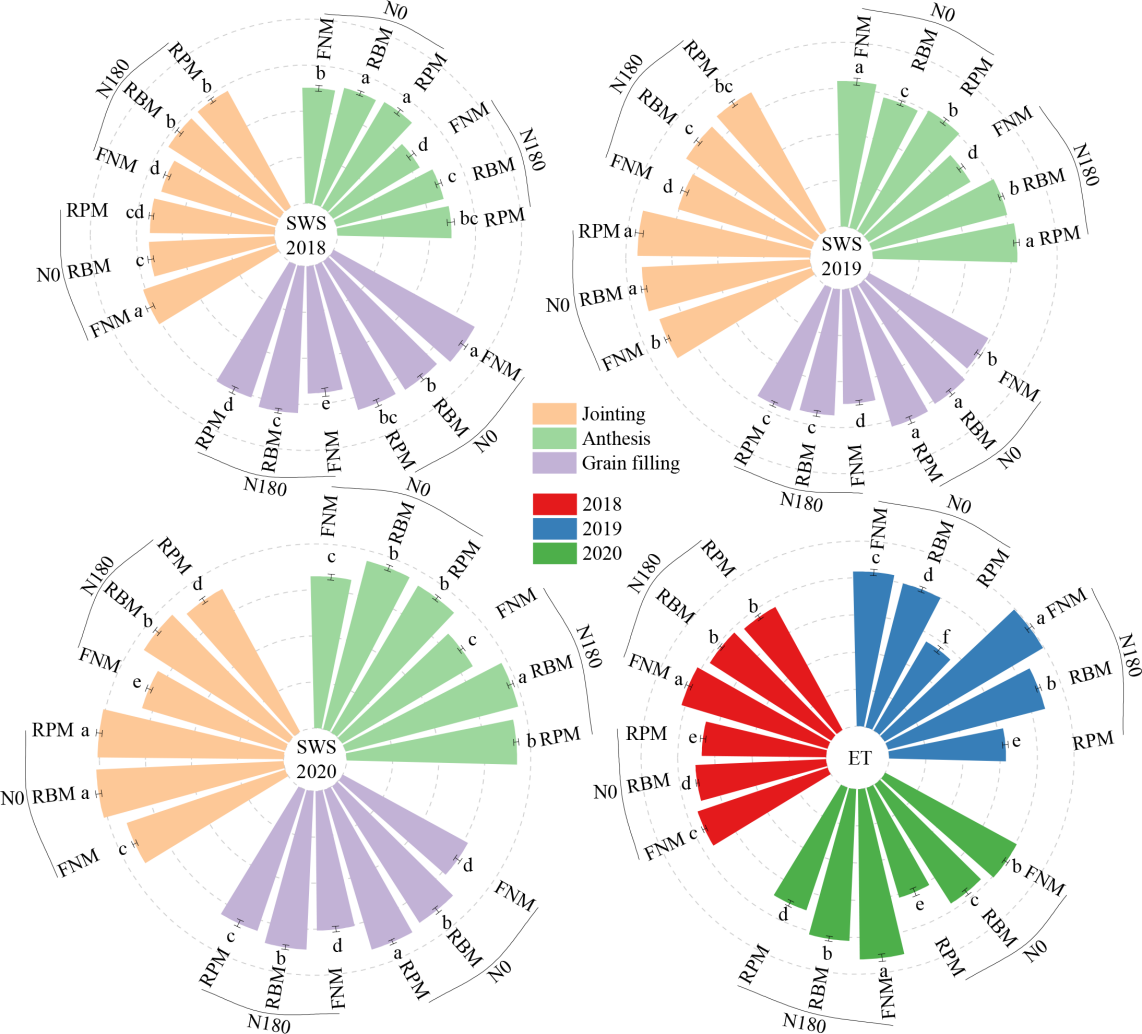
Soil water storage (SWS) in the 0–100 cm soil layer and evapotranspiration (ET) for maize during the growing seasons of 2018, 2019, and 2020. N0 and N180 represent the N application rates of 0 and 180 kg ha^–1^, respectively. FNM, RBM, and RPM represent flat planting without mulching, ridge-furrow mulching with biodegradable film, and ridge-furrow mulching with plastic film, respectively. Vertical bars and lowercase letters above the bars represent the standard error and significant differences between the treatments at the *P* < 0.05 level, respectively.

Film mulching under different nitrogen fertilization rates significantly increased SWS during the 2018, 2019, and 2020 seasons, except at the stages of jointing and grain-filling in 2018 under N0, and at the anthesis stage during 2019 under N0 (**Figure 2**). Compared with FNM, the average SWS in 2018, 2019, and 2020 increased remarkably (*P* < 0.05; the same applies to all differences in the following) by 10.0%, 9.5%, and 14.5%, respectively, under RBM, and by 10.1%, 12.5%, and 11.5% under RPM. Thereby, compared with FNM, in 2018, 2019, and 2020, the ET was 5.1%, 7.8%, and 8.1% lower, respectively, under RBM, and 8.7%, 34.8%, and 23.7% lower under RPM (**Figure 2**). The effect of nitrogen fertilization on SWS varied among the three growing seasons. In particular, compared with N0, the SWS in 2018, 2019, and 2020 was 16.2%, 15.9%, and 6.6% (except in the anthesis) lower, respectively, with N180 under FNM, but 5.8% (except for jointing), 10.0% (except in the anthesis), and 8.4% (only in the jointing stage) lower under RBM, and 7.2% (except in the jointing), 9.6% (except in the anthesis), and 11.5% (except in the anthesis) lower under RPM. However, compared with N0, the ET with N180 was 14.5%, 8.9%, and 12.9% higher under FNM, RBM, and RPM, respectively.

**Figure 3:**
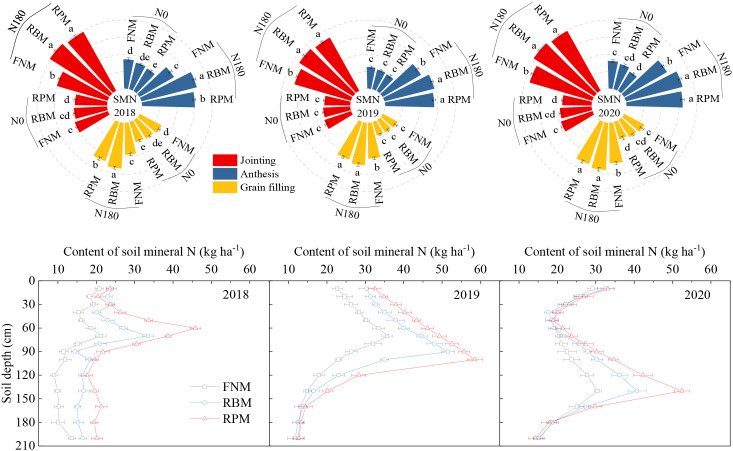
Content of soil mineral nitrogen (SMN) in 0–100 cm soil layer and distribution of soil mineral nitrogen at harvest under N application rates of 180 kg ha^–1^ for maize during the growing seasons of 2018, 2019, and 2020. N0 and N180 represent the N application rates of 0 and 180 kg ha^–1^, respectively. FNM, RBM, and RPM represent flat planting without mulching, ridge-furrow mulching with biodegradable film, and ridge-furrow mulching with plastic film, respectively. Bars and lowercase letters above the bars represent the standard error and significant differences between the treatments at the *P* < 0.05 level, respectively.

The impact of mulching coupled with nitrogen fertilization on SMN varied among stages in the three seasons (**Figure 3**). For instance, compared with FNM, the SMN was 6.8% and 12.6% lower under RBM and RPM, respectively, with N0, but 23.0% and 16.0% higher with N180. However, SMN increased significantly with nitrogen fertilization under all of the different mulching treatments. Compared with N0, the SMN was 78.2%, 131.6%, and 133.4% higher with N180 under FNM, RBM, and RPM, respectively. Furthermore, under N180, SMN was mainly concentrated in 40–90 cm, 30–120 cm, and 100–160 cm soil layers at harvest during the three seasons (**Figure 3**). In the main distribution depth, compared with FNM, the SMN in 2018 was 42.1% and 100.8% higher under RBM and RPM, respectively, 37.4% and 62.6% higher in 2019, and 25.7% and 48.7% higher in 2020.

During the growing seasons of 2018, 2019, and 2020, film mulching under different nitrogen fertilization rates decreased the SOM contents at the harvest by 25.4%, 9.9% (except under N180 in 2019), and 39.6%, respectively, compared with the SOM content before sowing in 2018 (**Table 1**). SOM only increased significantly by 7.6% and 11.5% under RBM and RPM with N0, respectively, in 2019; however, compared with N0, SOM only increased by 21.7%, 17.8%, and 14.3% under FNM, RBM, and RPM with N180, respectively, in 2019.

**Table 1:** The content of soil organic matter (SOM) in the 0–40 cm soil layer at the harvest during the maize seasons of 2018, 2019, and 2020.

Treatment		SOM (g/kg)			
		Pre-sowing	Growing season		
			2018	2019	2020
N0	FNM	18.7	15.0 a	15.7 c	13.0 a
	RBM	18.4	12.4 c	16.9 b	12.5 a
	RPM	18.5	14.3 ab	17.5 b	12.1 a
N180	FNM	18.2	13.7 b	19.1 a	10.8 b
	RBM	18.4	12.5 c	19.9 a	9.2 c
	RPM	18.3	14.5 ab	20.0 a	9.2 c

The N0 and N180 represent the N application rates of 0 and 180 kg ha^–1^, respectively. FNM, RBM, and RPM represent flat planting without mulching, ridge-furrow mulching with biodegradable film, and ridge-furrow mulching with plastic film, respectively. Values followed by different lowercase letters indicate significance at *P* < 0.05.

### 3.3 Dynamics of maize leaf area index and aboveground dry matter accumulation

**Figure 4:**
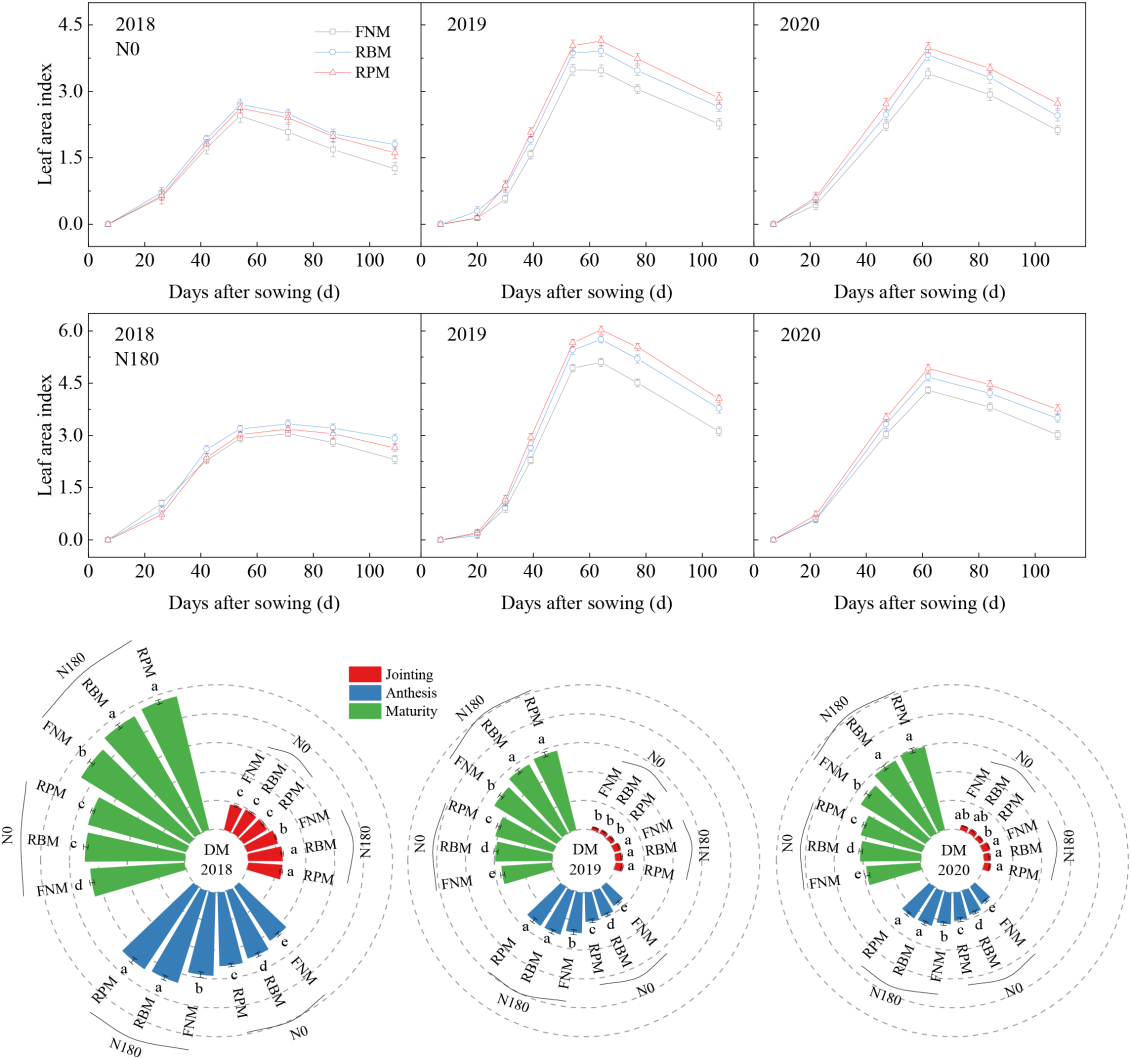
Leaf area index and aboveground dry matter of maize during the growing seasons of 2018, 2019, and 2020. N0 and N180 represent the N application rates of 0 and 180 kg ha^–1^, respectively. FNM, RBM, and RPM represent flat planting without mulching, ridge-furrow mulching with biodegradable film, and ridge-furrow mulching with plastic film, respectively. Vertical bars and lowercase letters above the bars represent the standard error and significant differences between the treatments at the *P* < 0.05 level, respectively.

The LAI peaked at around 60 days after sowing and then decreased across the three seasons (**Figure 4**). Compared with FNM, the LAI under RBM and RPM increased significantly during the period from the peak (around 60 days after sowing) to the harvest by 18.7% and 21.7% under N0, respectively, and by 14.9% and 17.2% under N180. Furthermore, compared with N0, the LAI under N180 was 47.5%, 42.7%, and 41.6% higher across the three growing seasons in 2018, 2019, and 2020, respectively.

Film mulching and N fertilization remarkably increased the dry matter (DM) during the growing seasons in 2018, 2019, and 2020 (except in the jointing stage) (**Figure 4**). Compared with FNM, the DM was 12.0% and 20.0% higher under RBM and RPM with N0, respectively, and 9.6% and 13.0% higher with N180. In addition, compared with N0, the DM was 49.9%, 43.6%, and 40.2% higher under N180 during the seasons of 2018, 2019, and 2020, respectively.

### 3.4 Reactive nitrogen emissions

**Figure 5:**
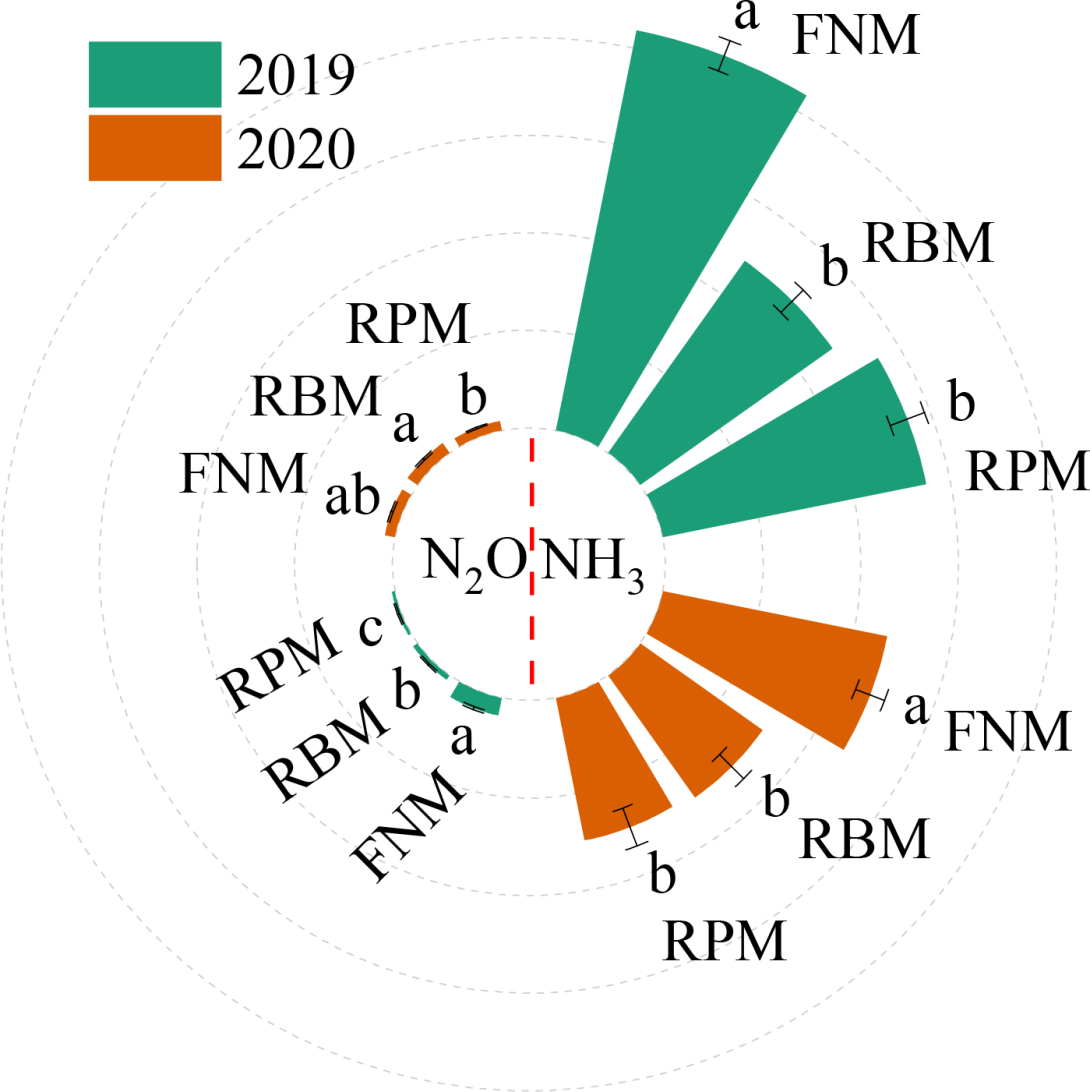
The amounts of NH_3_ volatilization and N_2_O emission under the N application rates of 180 kg ha^–1^ during the maize growing seasons of 2019 and 2020. FNM, RBM, and RPM represent flat planting without mulching, ridge-furrow mulching with biodegradable film, and ridge-furrow mulching with plastic film, respectively. Vertical bars and lowercase letters above the bars represent the standard error and significant differences between the treatments at the *P* < 0.05 level, respectively.

Film mulching under N application at 180 kg ha^–1^ significantly affected reactive N emissions (**Figure 5**). In particular, compared with FNM, the NH_3_ volatilization amount throughout the whole season was 38.3% and 35.3% lower under RBM and RPM, respectively. However, compared with FNM, the N_2_O emissions throughout the whole season under RBM and RPM were only significantly lower in 2019 when they were 69.4% and 82.3% lower, respectively.

### 3.5 Maize yield and water and nitrogen use efficiencies

Compared with FNM, the yield was 6.2% and 8.4% higher under RBM and RPM with N180, respectively, and the WUE was 17.3% and 44.4% higher (**Table 2**). Moreover, compared with FNM, during the three seasons, WUE was 32.3% higher under RPM with N0. Compared with FNM, during the three seasons, the NHI was 6.6% and 7.7% higher under RBM and RPM with N0, respectively, and 11.8% and 13.9% higher with N180. However, compared with FNM, the NUE was only 5.3% and 3.4% higher under RBM and RPM with N0, respectively, and 6.7% (except in the 2020 season) and 3.7% (only in the 2019 season) higher with N180. During the seasons of 2018, 2019, and 2020, Compared with N0, the yield, WUE, and NUE were 46.7%, 28.4%, and 9.0% higher under FNM with N180, respectively, 52.1%, 39.6%, and 9.0% higher under RBM, and 57.1%, 38.9%, and 7.6% higher under RPM, but the NHI was 16.5%, 12.5%, and 11.7% lower under FNM, RBM, and RPM.

**Table 2:** Grain yield, water use efficiency (WUE), nitrogen use efficiency (NUE), and nitrogen harvest index (NHI) for maize during the seasons of 2018, 2019, and 2020.

Year	Treatment		Yield (kg/hm^2^)	WUE (kg/hm^2^/mm)	NUE (kg/kg)	NHI (%)
2018	N0	FNM	6196 c	13.9 d	52.9 c	54.3 b
		RBM	6247 c	14.8 cd	53.9 c	60.2 a
		RPM	6264 c	15.6 c	52.7 c	60.4 a
	N180	FNM	9205 b	18.7 b	55.9 b	46.7 d
		RBM	9671 a	20.7 a	60.0 a	52.9 c
		RPM	9709 a	21.4 a	56.9 b	53.9 b
2019	N0	FNM	5462 d	10.9 d	48.7 d	60.6 b
		RBM	5726 c	11.9 d	52.1 c	63.5 a
		RPM	5547 cd	16.6 b	50.8 c	64.7 a
	N180	FNM	8053 b	13.5 c	54.2 b	49.0 e
		RBM	8803 a	16.8 b	57.5 a	53.7 d
		RPM	9058 a	24.0 a	56.2 a	55.0 c
2020	N0	FNM	5974 c	12.3 d	51.6 d	66.2 b
		RBM	6068 c	13.2 d	53.5 c	69.0 a
		RPM	6038 c	16.3 c	52.9 c	69.6 a
	N180	FNM	8604 b	15.6 c	56.8 a	55.3 e
		RBM	8958 a	18.2 b	56.4 a	62.2 d
		RPM	9232 a	22.0 a	55.1 ab	63.0 c

The N0 and N180 represent the N application rates of 0 and 180 kg ha^–1^, respectively. FNM, RBM, and RPM represent flat planting without mulching, ridge-furrow mulching with biodegradable film, and ridge-furrow mulching with plastic film, respectively. Values followed by different lowercase letters indicate significance at *P* < 0.05.

### 3.6 Effects of mulching on maize production and associated pathways

TOPSIS analysis showed that RBM and RPM were more effective for maize production in the subhumid drought-prone study area than FNM under different nitrogen fertilization rates during the seasons of 2018, 2019, and 2020, and their effects were similar, especially under N180 (**Supplementary Table 1**). PCA indicated that the first two main components explained 84.3% of the total difference (PC1 explained 70.2% and PC2 explained 14.1%) (**Supplementary Figure 1**). Furthermore, under RBM and RPM, SMN and plant growth were the key factors related to an increase in the maize yield and resource utilization, and there was no great difference between RBM and RPM.

**Figure 6:**
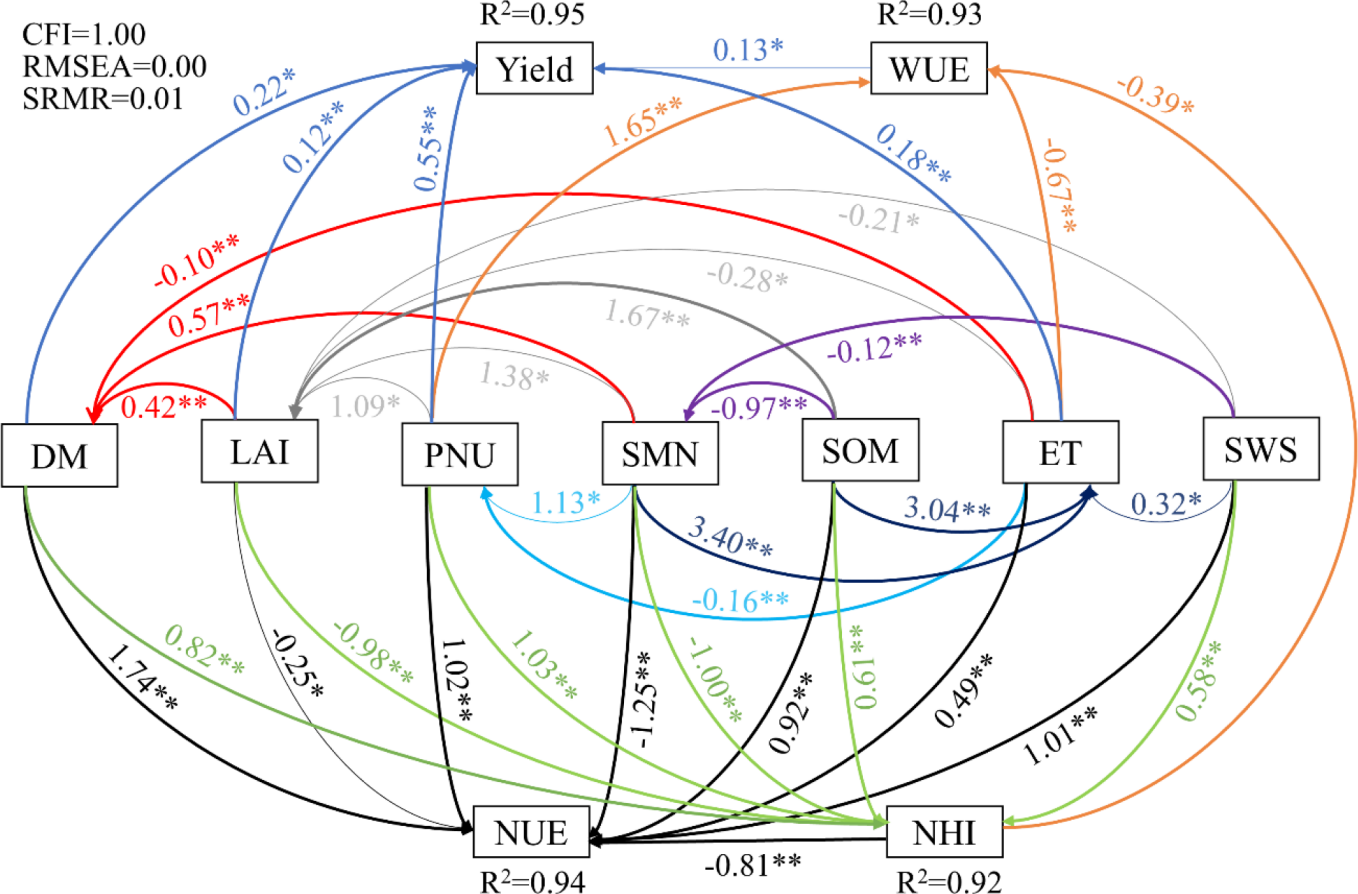
Structural equation modeling for yield, WUE, NUE, and NHI of maize under film mulching. The DM, LAI, and PNU are aboveground dry matter, leaf area index, and plant nitrogen uptake, respectively. SMN and SWS are the mean content of soil mineral nitrogen and mean soil water storage across the main growth stages, respectively. SOM and ET are the content of soil organic matter and evapotranspiration, respectively. WUE, NUE, and NHI are water use efficiency, nitrogen use efficiency, and nitrogen harvest index, respectively. Numbers at arrows are standardized path coefficients. * and ** indicate significance at *P* < 0.05 and *P* < 0.01, respectively. R^2^ values on top of response variables indicate the proportion of variation explained by the environmental factors and the constructed relationships. The CFI, RMSEA, and SRMR represent comparative fit index, rooted mean square error of approximation, and standardized root mean square residual, respectively.

SEM showed that the maize yield was directly and positively influenced by DM, LAI, PNU, and ET, and their effects explained 95% of the total variance in the yield (**Figure 6**). Moreover, SOM positively affected LAI, and LAI and SMN increased the yield by positively influencing DM and PNU. WUE was directly and positively affected by PNU but negatively influenced by ET and NHI, and they together explained 93% of the total variance in WUE. In addition, LAI and SMN positively affected WUE by positively influencing PNU, whereas SMN, SOM, and SWS negatively affected WUE by positively influencing ET. NUE was directly and positively affected by DM, PNU, SOM, ET, and SWS, but negatively affected by LAI and SMN, and they together explained 94% of the total variance in NUE. Furthermore, both LAI and SMN positively affected NUE by positively influencing DM and PNU. SOM, SMN, and SWS also positively affected NUE by positively influencing ET. The effects of mulching on NHI and the associated pathways were similar to those for NUE (except for ET).

## 4 Discussion

### 4.1 Effects of mulching coupled with N fertilization on soil fertility

RFFM can collect rainfall and prevent soil water losses to improve the soil moisture content (Daryanto et al., 2017). Previous research has indicated that RFFM improved SWS and decreased ET (Fang et al., 2022a; Shangguan et al., 2024). Similar results were obtained in present study, but the SWS was opposite under RBM and RPM with N0 at the stages of jointing and grain-filling in the 2018 season and the anthesis stage in the 2019 season, possibly due to the low precipitation during these growth periods (**Figure 1**), and film mulching intensified the consumption of soil water by maize (Liu et al., 2022). Furthermore, the SWS under RBM was comparable to that under RPM, as also shown by Gu et al. (2021). In general, nitrogen fertilization indirectly affects the soil moisture content via its influence on plant growth (Wang et al., 2019). Here, nitrogen fertilization mainly decreased the soil moisture but increased ET, and its effect on SWS was related to precipitation under film mulching, especially in the 2020 season, possibly because nitrogen fertilization rose the consumption of soil moisture by promoting maize growth (**Figure 5**), and the distribution and infiltration of soil moisture were affected when it was combined with film mulching (Chen et al., 2019).

SMN can be directly utilized by maize, affecting its growth and yield. In the present study, the SMN contents decreased under RBM and RPM with N0 but increased with N180, possibly because mulching increased the absorption of SMN by plants due to improved plant growth, and film mulching under N180 also reduced the loss of SMN (Fang et al., 2021b; Zhang et al., 2025). Nitrogen fertilization increased SMN, especially coupled with film mulching, as found in former research (Wang et al., 2019; Song et al., 2024). Mulching can maintain soil fertility, but it may also accelerate nitrogen leaching. In the present study, at the harvest in 2018, SMN was mainly concentrated in the 40–90 cm soil depth and deep leaching tended to occur throughout the subsequent growing seasons, which could be explained by the high rainfall in the 2019 and 2020 seasons, especially in 2020 (**Figure 1**). Furthermore, the distribution depth of SMN was similar under the different film mulching patterns, thereby indicating that film mulching did not accelerate the leaching of nitrogen into the deep soil (Lai et al., 2024).

SOM is vital for soil fertility. Yang et al. (2024) indicated that RPM significantly increased the soil organic carbon content (which can be transformed into SOM). In the present study, SOM generally declined during the growing season and varied among treatments because there was no organic fertilizer was added in this study, and the main source of SOM was the maize root system. In addition, the SOM contents did not differ significantly under RBM and RPM, as found in former research (Bo et al., 2024; Zhang et al., 2025). The result suggested that planting with RFFM for a long time would require extra organic material inputs to maintain the SOM content.

### 4.2 Effects of mulching coupled with N fertilization on maize growth

RFFM and N fertilization change the soil properties, affecting maize growth. Here, RBM and RPM enhanced the LAI from about 50 days after sowing because the LAI is related to the nutrients obtained by plants, and the nutrient demand of maize increased sharply from about 50 days after sowing due to its vigorous growth (Li and Mu, 2021; Zhang et al., 2025). Furthermore, RBM and RPM increased DM during the growing seasons of 2018, 2019, and 2020, as found in previous research (Chen et al., 2021; Fang et al., 2022b). Nevertheless, RBM and RPM decreased DM during the jointing stage in the 2020 season, especially when combined with nitrogen fertilization, possibly due to the higher rainfall during the jointing stage in the 2020 season, reducing soil aeration under RBM and RPM (**Figure 1**), thereby affecting maize root growth (Li et al., 2020; Fang et al., 2022a).

### 4.3 Effects of mulching on reactive nitrogen emissions

RFFM reduces the area of bare soil, thereby diminishing soil-atmosphere gas exchange. However, RFFM alters factors such as soil moisture, heat, and nutrients, which may increase reactive nitrogen emissions from fields (Zheng et al., 2021). Here, the peak periods for NH_3_ and N_2_O emissions mainly occurred within 2 weeks after sowing, and NH_3_ volatilization and N_2_O emissions decreased significantly (except in the 2020 season) under RBM and RPM with N180, as was also obtained in former research (Zhang et al., 2021; Fang et al., 2022b). The different N_2_O emissions in the 2020 season could be explained by the greater rainfall after sowing (**Figure 1**), enhancing N_2_O emissions (Wang et al., 2024).

### 4.4 Effects of mulching coupled with N fertilization on yield and resource utilization

RFFM and N fertilization can improve the soil nutrient contents and plant growth, affecting the maize yield and resource utilization. Previous research reported that RFFM coupled with N fertilization increased maize production (Lai et al., 2024; Zhang et al., 2025), and the impact of biodegradable film was inferior or comparable to that of polyethylene film (Fang et al., 2022a; Shangguan et al., 2024; Xiong et al., 2025). In present study, similar results were also found regarding the effects of RBM and RPM under N180 on the maize yield, WUE, NUE, and NHI. However, RBM and RPM did not always increase WUE and NUE during the three growing seasons, especially NUE in the 2020 season, possibly due to the higher precipitation in 2019 and 2020 (**Figure 1**), especially in 2020, replenishing the soil moisture but also readily leaching mineral N (Zhang et al., 2025) to affect ET and PNU. N fertilization increased the yield, WUE, NUE, and NHI within certain ranges (Du et al., 2024). However, N fertilization decreased NHI during the three growing seasons in the present study, possibly because applying N180 exceeded the reasonable N application amount, as RFFM can reduce the need for applying N (Xiong et al., 2025).

Some previous studies have explored the mechanisms associated with the increases in production and efficiency under RFFM. For instance, Zhang et al. (2025) identified soil properties, such as the available phosphorus and urease and catalase activities, as key growth, yield, and WUE determinants in maize. However, it is not clear how RBM and RPM might increase the yield and resource utilization of maize. Here, SMN and plant growth were identified as the main factors that improved the yield, WUE, and N utilization in maize under RBM and RPM. The yield was directly and positively affected by DM, LAI, PNU, and ET, and SMN and SOM increased the yield through influencing plant growth, as also shown by Xiong et al. (2025). WUE was directly and positively affected by PNU but negatively influenced by ET and NHI, and LAI and SMN increased WUE by affecting PNU because promoting maize growth is conducive to increasing the yield, and reducing the consumption of water can enhance WUE according to Equation 3 (Jing et al., 2024). NUE was directly and positively affected by DM, PNU, SOM, ET, and SWS. Furthermore, LAI and SMN increased NUE by affecting DM and PNU, but SOM, SMN, and SWS increased NUE by influencing ET because NUE is related to the yield and PNU, and RFFM affects the soil nutrient distribution and conversion of soil carbon and N (Yang et al., 2024) to influence uptake and redistribution of N by plants (Lai et al., 2024).

## 5 Conclusions

In conclusion, RBM and RPMenhanced maize productivity by improving soil water storage and mineral nitrogen content while decreasing ET and reactive N emissions. Despite a tendency to increase the consumption of SOM, both RBM and RPM with N180 significantly promoted plant growth. The pathways of soil fertility and maize growth on yield and utilization of water and N were different, but yield and WUE increased under RBM and RPM with N180, and NHI and NUE increased regardless of fertilization. Moreover, the effect of RBM was similar to that of RPM. Therefore, the results suggested that RBM could replace RPM in maize production.

## Data Availability

The original contributions presented in the study are included in the article/**Supplementary Material**. Further inquiries can be directed to the corresponding authors.
