# Supplementary material for: Biodegradable film: a sustainable alternative to polyethylene film for Loess Plateau maize production
Source: Front Plant Sci. 2026 Mar 11;17:1789837. doi: 10.3389/fpls.2026.1789837 (PMC13015842; doi:10.3389/fpls.2026.1789837)
Supplement: Supplementary file 1 [file Table1.docx]

**Table S1**

Comprehensive effect evaluation for maize production with data of SOM, SMNS, SMNA, SWS, ET, reactive nitrogen emissions, aboveground dry matter, LAI, grain yield, WUE, and NUE under different film mulching and nitrogen fertilization.

| Treatment | | 2018 | |  | 2019 | |  | 2020 | |
| --- | --- | --- | --- | --- | --- | --- | --- | --- | --- |
|  |  | Proximity | Rank |  | Proximity | Rank |  | Proximity | Rank |
| N0 | FNM | 0.126 | 6 |  | 0.123 | 6 |  | 0.115 | 6 |
|  | RBM | 0.139 | 5 |  | 0.138 | 5 |  | 0.141 | 5 |
|  | RPM | 0.160 | 3 |  | 0.157 | 3 |  | 0.156 | 4 |
| N180 | FNM | 0.150 | 4 |  | 0.154 | 4 |  | 0.172 | 3 |
|  | RBM | 0.201 | 2 |  | 0.204 | 2 |  | 0.202 | 2 |
|  | RPM | 0.223 | 1 |  | 0.225 | 1 |  | 0.214 | 1 |

The N0 and N180 represent the N application rates of 0 and 180 kg ha^–1^, respectively. FNM, RBM, and RPM represent flat planting without mulching, ridge-furrow mulching with biodegradable film, and ridge-furrow mulching with plastic film, respectively. SOM is the content of soil organic matter (SOM) in the 0–40 cm soil layer at the harvest. SMNS and SMNA are the content of soil mineral nitrogen in the 0–100 cm soil layer and the distribution of soil mineral nitrogen at harvest under N application rates of 180 kg ha^–1^, respectively. SWS is oil water storage in the 0-100 cm soil layer. ET is evapotranspiration at each season. WUE and NUE are water use efficiency and nitrogen use efficiency, respectively.

SOM

NHI

SWS

LAI

ET

DM

NUE

Yield

PNU

SMN

WUE

Fig. S1. Principal component analysis of maize production under film mulching and nitrogen fertilization. RBM and RPM represent ridge-furrow mulching with biodegradable film and ridge-furrow mulching with plastic film, respectively. SOM is the content of soil organic matter. SWS and SMN are the mean soil water storage and mean content of soil mineral nitrogen across the main growth stages, respectively. ET is evapotranspiration. LAI is the leaf area index. DM is aboveground dry matter. PNU is plant nitrogen uptake. NUE and NHI are nitrogen use efficiency and nitrogen harvest index, respectively. WUE is water use efficiency.
